# Integrated Positioning for Coal Mining Machinery in Enclosed Underground Mine Based on SINS/WSN

**DOI:** 10.1155/2014/460415

**Published:** 2014-01-21

**Authors:** Qigao Fan, Wei Li, Jing Hui, Lei Wu, Zhenzhong Yu, Wenxu Yan, Lijuan Zhou

**Affiliations:** ^1^Key Laboratory of Advanced Process Control for Light Industry, Ministry of Education, Jiangnan University, Wuxi 214122, China; ^2^School of Mechanical and Electrical Engineering, China University of Mining and Technology, Xuzhou 221008, China; ^3^Avlc Radar and Avionics Institute, Wuxi 214122, China

## Abstract

To realize dynamic positioning of the shearer, a new method based on SINS/WSN is studied in this paper. Firstly, the shearer movement model is built and running regularity of the shearer in coal mining face has been mastered. Secondly, as external calibration of SINS using GPS is infeasible in enclosed underground mine, WSN positioning strategy is proposed to eliminate accumulative error produced by SINS; then the corresponding coupling model is established. Finally, positioning performance is analyzed by simulation and experiment. Results show that attitude angle and position of the shearer can be real-timely tracked by integrated positioning strategy based on SINS/WSN, and positioning precision meet the demand of actual working condition.

## 1. Introduction

With the development of the coal exploitation technology and the improvement of coalmine safety requirements, man less coalmine extraction becomes a trendy. Man's less extraction needs mechanization and automation of mining equipment. Mechanical and electrical equipment on the comprehensive mechanized working face includes the shearer, the hydraulic support, and the flexible scraper conveyor (which are called the three machines). Collaborative working of the three machines depends on machinery-tracked automation of the hydraulic support, because the shearer dynamic position determines the machinery-tracked automation of the hydraulic support [1, 2]. As the shearer dynamic position precision is helpful for hydraulic support automation input, as well as the shearer drum height adjusting, this paper focuses on shearer dynamic positioning.

A typical shearer such as SL500 can be as long as 16 m and max cutting height in excess of 5.5 m. The traveling speeds of shearers can vary by about 0~25.5 m/min. However, the speeds of shearers are on the increase, and there is now a reported case of 30 m/min. Shearer dynamic positioning precision is difficult, because the shearer movement parameters vary (such as the velocity, the acceleration, and the position) real-timely. Also, random vibration and coal rock properties disturb the movement parameters.

A gear counting method is proposed for shearer positioning [3]. The principle is the shearer distance is calculated by multiplying the gear revolutions by the gear perimeter, and the gear revolutions are obtained by counting sensors. This method is simple and easy. However, the gear counting cannot indicate the directions, as the shearer walking directions includes two aspects: the direction along the working face and the vertical direction. Thus, positioning error is produced. Another problem unsolved is error accumulation of the gear counting. An infrared positioning system is established for the shearer [4]. An infrared emission module was installed on the shearer and a receiver module on the hydraulic support to receive signal. The infrared emission module launches wide-angle pulse to the receiver module, then the shearer position can be obtained by calculating the received signal strength. This method is feasible, but inherent error exists, because the system obtains the shearer position quantitatively. Another positioning method based on wireless sensor networks is described for the shearer [5]. The shearer distance was calculated through a received signal-strength attenuation model in underground. Dai and Akyildiz [6] and Vuran and Akyildiz [7] investigated a communication channel model which was used in underground wireless sensor network. As RF signal attenuation index in the underground depends on wireless channel model, this method has not been put into practical use. Reid and Hainsworth [8] researched a shearer positioning system using inertial navigation, in which attitude and location information of the shearer are obtained by the gyroscope and linear accelerometers. Fang et al. [9] built a dynamic model of the shearer and investigated a shearer positioning method theoretically using strapdown inertial navigation. But the accumulative error and sensor drift exist.

From the above discussion we know that researches on shearer positioning have been done. Judging the advantages and disadvantages, this paper next will discuss a strapdown inertial navigation positioning system in the underground for the shearer. This paper first established the shearer movement model and analyzed the relationship between the shearer position and the automatic machinery-tracked system and then adopt a strategy to achieve the shearer dynamic position, and the strategy was called strapdown inertial navigation system (SINS) attitude and position updating based on quaternion. Since the coal mine is closed, SINS error cannot be corrected by global position system (GPS). On this basis, a new recalibration scheme based on wireless sensor networks (WSN) is proposed in this paper. It is concluded that this scheme is useful for the SINS shearer positioning system.

## 2. Movement Model of the Shearer

**Figure 1:**
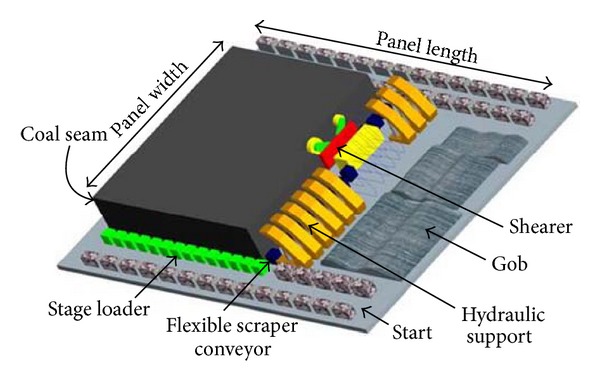
The coal mine working face by 3D schematic.

As shown in Figure 1, the shearer walks on the flexible scraper conveyor and cuts the coal; the hydraulic support supports the roof. Given the direction of the shearer, the walking distance of the shearer is

(1) $$\begin{array}{c} L=\overset{t}{\underset{0}{\int }}v_{q}dt+l_{0}, \end{array}$$

where *v*_*q*_ is the shearer haulage speed and *l*_0_ is the shearer displacement at the initial time. The number of the hydraulic support is

(2) $$\begin{array}{c} K=\mathrm{int}\left|\frac{L}{A}\right|=\mathrm{int}\left|\frac{\left(\int_{0}^{t}v_{q}+l_{0}\right)}{A}\right|, \end{array}$$

where *A* is hydraulic support frame spacing.

**Figure 2:**
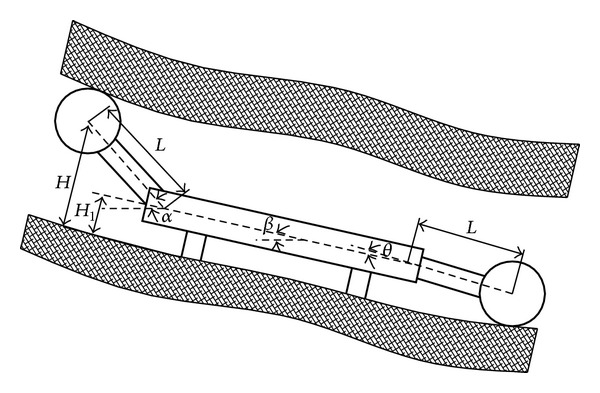
Shearer uplinking cutting schematic.

In the working face, shearer drum automatic height adjusting closely relates to the shearer attitude [10]. As shown in Figure 2, angle between shearer and horizon line is *β* (which is also called pitch angle); angles between two rock arms and horizon line are, respectively, *α* and *θ*; the arm is *L*; then the shearer drum height *H* is

(3) $$\begin{array}{c} H=H_{1}+L\times \sin\left(\alpha -\beta \right). \end{array}$$

**Figure 3:**
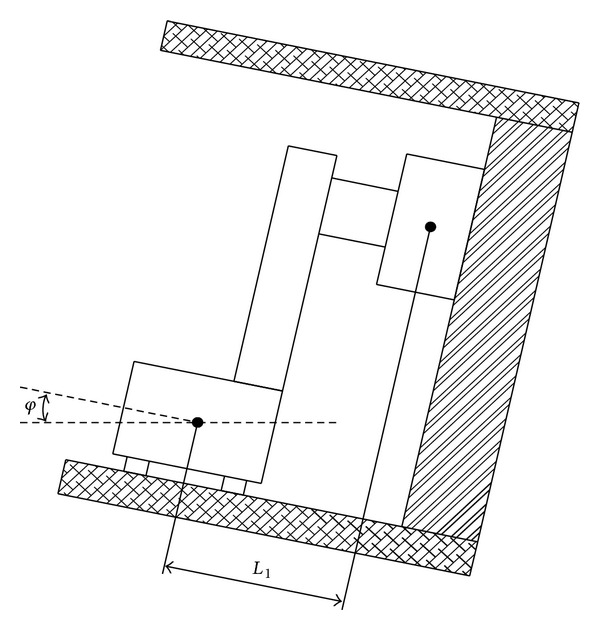
Shearer side view along working face pushing direction.

Also, the shearer has tilt angle along working pushing direction, as shown in Figure 3. The tilt angle is *φ* (which is also called roll angle); center distance between Shearer drum and Shearer base is *L*_1_; then we get

(4) $$\begin{array}{c} \Delta H=L_{1}\times \tan\left(\varphi \right). \end{array}$$

Combining (3), we get the shearer drum height *H*′:

(5) $$\begin{array}{c} H^{\prime }=H-\Delta H, \end{array}$$

(6) $$\begin{array}{c} H^{\prime }=H_{1}+L\times \sin\left(\alpha -\beta \right)-L_{1}\times \tan\left(\varphi \right). \end{array}$$

As shown in (6), there are direct connections between the shearer drum height and the shearer attitude angle. So the shearer dynamic positioning system is also very important for shearer automatic adjusting height.

## 3. Shearer Positioning Strategy Based on SINS/WSN

Positioning strategy based on SINS/WSN includes the following points. Angular velocities from the gyroscopes construct an attitude matrix, and the attitude matrix includes the shearer attitude and course information. And acceleration of the shearer from the linear accelerometers is transformed from the carried coordinate to the geographic coordinate [11]. Then by navigation solution and external calibration, we obtain the shearer position information.

### 3.1. Attitude Solution

As described above, the shearer has two tilt angles: pitch angle *β* and roll angle *φ*. Another angle *α* called yawing angle exists when the shearer is obliquely cutting and feeding. *α* is the angle between the shearer plane projection and the northern direction. *β*, *φ*, and *α* are called the shearer attitude angles. As in SINS, position relation between the carrier attitude and the course direction is the angle between the body reference frame (shorted for *OX*_*b*_ *Y*_*b*_ *Z*_*b*_) and the geographic coordinate (shorted for *OX*_*n*_ *Y*_*n*_ *Z*_*n*_) [12].

Given a sample time Δ*t*, outputs of the gyroscope are angular velocity *ω*_*ib*_^*b*^ in *X*_*b*_ *Y*_*b*_ *Z*_*b*_, transforming *ω*_*ib*_^*b*^ into *ω*_*nb*_^*b*^ in *OX*_*n*_ *Y*_*n*_ *Z*_*n*_:

(7) $$\begin{array}{c} \omega_{nb}^{b}=\omega_{ib}^{b}-\omega_{ie}^{b}-\omega_{en}^{b}, \end{array}$$

where *ω*_*ib*_^*b*^ is angular velocity in *OX*_*b*_ *Y*_*b*_ *Z*_*b*_, which is relative to *OX*_*i*_ *Y*_*i*_ *Z*_*i*_; *ω*_*ie*_^*b*^ is angular velocity of rotation of the earth in *OX*_*b*_ *Y*_*b*_ *Z*_*b*_, which is relative to *OX*_*e*_ *Y*_*e*_ *Z*_*e*_; *ω*_*en*_^*b*^ is rotating angular velocity in *OX*_*b*_ *Y*_*b*_ *Z*_*b*_, which is relative to *OX*_*e*_ *Y*_*e*_ *Z*_*e*_; *ω*_*nb*_^*b*^ is angular velocity in *OX*_*b*_ *Y*_*b*_ *Z*_*b*_; which is relative to *OX*_*n*_ *Y*_*n*_ *Z*_*n*_.

We get *ω*_*ie*_^*b*^:

(8) $$\begin{array}{c} \omega_{ie}^{b}=C_{n}^{b}{\left[\begin{array}{ccc} 0 & \omega_{ie}\cos L & \omega_{ie}\sin L \end{array}\right]}^{T}, \end{array}$$

where *C*_*n*_^*b*^ is the attitude matrix at the initial attitude angle; it is revised by quaternion when the shearer rotates about the *X*_*i*_ axis in *OX*_*i*_ *Y*_*i*_ *Z*_*i*_; *ω*_*ie*_ is angular velocity of rotations of the earth; *ω*_*ie*_ = 15.041 deg/h.

And *ω*_*en*_^*b*^ in (7) is

(9) $$\begin{array}{c} \omega_{en}^{b}=C_{n}^{b}{\left[\begin{array}{ccc} -\dot{L} & \dot{\lambda }\cos L & \dot{\lambda }\sin L \end{array}\right]}^{T}, \end{array}$$

where *L* is the latitude and *λ* is the longitude. Here we introduce the quaternion calculation. As the quaternion matrix is *Q* = [*q*_0_ *q*_1_ *q*_2_ *q*_3_], derivation of *Q* is

(10) $$\begin{array}{c} \dot{Q}=\frac{1}{2}\Omega \cdot Q, \end{array}$$

where *Ω* is the antisymmetric matrix of *ω*_*nb*_^*b*^ and *ω*_*nb*_^*b*^ is constant in a sample time Δ*t*. Let *P* be *P* = *Ω* · Δ*t*; solving (10) by using the Picard iterative method, we can revise quaternion [13]:

(11) $$\begin{array}{c} Q\left(t+\Delta t\right)=\left(\cos\frac{\Delta \theta }{2}\cdot I+\frac{\sin\left(\Delta \theta /2\right)}{\Delta \theta }\cdot P\right)\cdot Q\left(t\right), \end{array}$$

where Δ*θ* is the real-time angular increment.

In (11), if giving initial value of *Q*(*t*) when *t* = 0, we can calculate *Q*(*t* + Δ*t*) at the next time *t* + Δ*t*. Based on the revised quaternion, updating the shearer attitude matrix *C*_*n*_^*b*^ in (9),

(12) Cnb=[C11C12C13C21C22C23C31C32C33]=[q02+q12−q22−q322(q1q2+q0q3)2(q1q3−q0q2)2(q1q2−q0q3)q02−q12+q22−q322(q2q3+q0q1)2(q1q3+q0q2)2(q2q3−q0q1)q02−q12−q22+q32].

Elements in (12) help to calculate the attitude angles of the shearer in real time:

(13) $$\begin{array}{c} \alpha =-\text{arctg }\left(\frac{C_{21}}{C_{22}}\right),\\ \beta =\arcsin\left(C_{23}\right),\\ \varphi =\text{ arctg }\left(-\frac{C_{13}}{C_{33}}\right), \end{array}$$

where *C*_21_, *C*_22_, *C*_23_, *C*_13_, and *C*_33_ are elements in (12).

### 3.2. Position Solution

Similarly, the velocity and the position resolving are calculated depending on linear accelerometers output. As the linear accelerometer outputs specific force vector *f* ^*b*^ in *OX*_*b*_ *Y*_*b*_ *Z*_*b*_, we transform *f* ^*b*^ into *f* ^*n*^ in *OX*_*n*_ *Y*_*n*_ *Z*_*n*_ [14, 15]:

(14) $$\begin{array}{c} f^{n}=C_{n}^{b}\cdot f^{b}. \end{array}$$

As in inertial navigation [16, 17],

(15) $$\begin{array}{c} {\dot{V}}^{n}=f^{n}-V-g^{n}, \end{array}$$

where *f* ^*n*^ is the shearer specific force vector in *OX*_*n*_ *Y*_*n*_ *Z*_*n*_, *f* ^*n*^ = [*f*_*E*_ *f*_*N*_ *f*_*U*_]^*T*^, *V* ^*n*^ is the shearer velocity, *V* ^*n*^ = [*V*_*E*_ *V*_*N*_ *V*_*U*_]^*T*^, *g* ^*n*^ is the gravitational acceleration, and *g* ^*n*^ = [0 0 −*g*]^*T*^.

Consider

(16) V=(2ωien+ωenn)Vn=[0(λ˙+2ωie)sinL−(λ˙+2ωie)cosL(λ˙+2ωie)sinL0−L˙(λ˙+2ωie)cosLL˙0] ×[VEVNVU].

Combining (15) and (16), *V*_*E*_, *V*_*N*_, and *V*_*U*_ in (16) are resolved and *V*_*E*_, *V*_*N*_, and *V*_*U*_ are the component of shearer velocity in *OX*_*n*_ *Y*_*n*_ *Z*_*n*_.

As

(17) $$\begin{array}{c} \dot{L}=\frac{V_{N}}{R_{N}+h},\\ \dot{\lambda }=\frac{V_{E}}{\left(R_{E}+h\right)\cos L},\\ \dot{h}=V_{U}, \end{array}$$

where *h* is the shearer height, *R*_*N*_ is the earth ellipsoid local meridian, and *R*_*E*_ is prime vertical curvature radius, given the initial latitude *L*(0), initial longitude *λ*(0), and initial height *h*(0), integrating (17), the shearer position updating formula is

(18) $$\begin{array}{c} L=\int \dot{L}dt+L\left(0\right),\\ \lambda =\int \dot{\lambda }dt+\lambda \left(0\right),\\ h=\int \dot{h}dt+h\left(0\right). \end{array}$$

**Figure 4:**
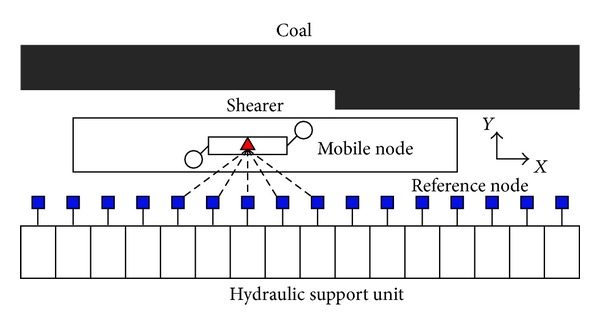
Diagram of node arrangement based on WSN on the fully mechanized coal face.

**Figure 5:**
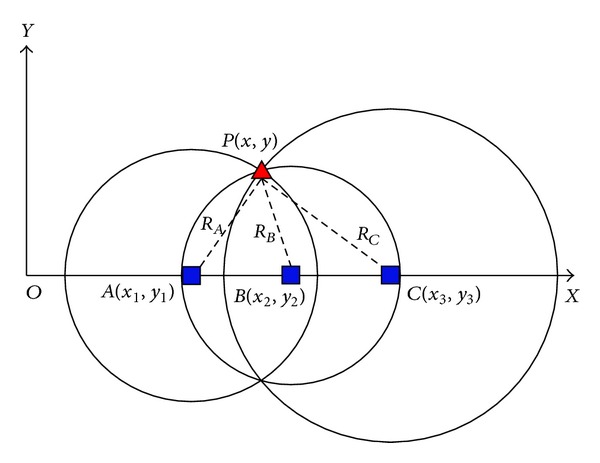
Location model diagram of the shearer based on WSN.

As low accuracy of the triaxial acceleration sensor results in serious drift of SINS during run time, we proposed indoor positioning method of WSN as a kind of compensation scheme [18, 19]. Node arrangement diagram of WSN on the fully mechanized coal face is shown in Figure 4, and positioning mathematical model is built, as shown in Figure 5.

Given the position line equation of WSN,

(19) $$\begin{array}{c} R_{A}=\sqrt{{\left(x-x_{1}\right)}^{2}+{\left(y-y_{1}\right)}^{2}},\\ R_{B}=\sqrt{{\left(x-x_{2}\right)}^{2}+{\left(y-y_{2}\right)}^{2}},\\ R_{C}=\sqrt{{\left(x-x_{3}\right)}^{2}+{\left(y-y_{3}\right)}^{2}}, \end{array}$$

(20) $$\begin{array}{c} R_{A}=cT_{oA},\\ R_{B}=cT_{oB},\\ R_{C}=cT_{oC}, \end{array}$$

where *T*_*oA*_, *T*_*oB*_, and *T*_*oC*_ are time difference of arrival of *A*(*x*_1_, *y*_1_), *B*(*x*_2_, *y*_2_) and *C*(*x*_3_, *y*_3_), respectively. Mobile node of shearer *P*(*x*, *y*) can be got by any two equations in (19). While in the actual working condition, *A*(*x*_1_, *y*_1_), *B*(*x*_2_, *y*_2_), and *C*(*x*_3_, *y*_3_) probably not in a straight line, we can obtain no fuzzy solution through three reference nodes.

**Figure 6:**
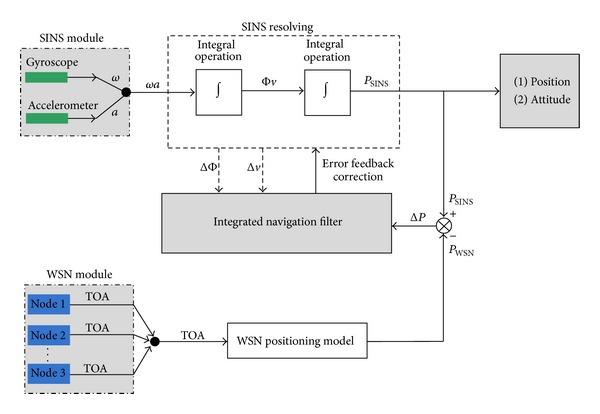
Coupled model diagram of SINS and WSN.

As SINS and WSN are two separate systems, coupling model is built in order to make the complementary advantages, as shown in Figure 6.

On the one hand, SINS outputs angular velocity *ω* and linear acceleration *α*. When the signal is integrated for one time, attitude angle Φ and velocity *v* can be obtained, and when the signal is integrated for two times, displacement *P*_SINS_ can be obtained. On the other hand, according to the time difference of arrival between mobile node and reference node, location of the shearer *P*_WSN_ is determined. Then Δ*P* can be got by value comparison between *P*_SINS_ and *P*_WSN_. Finally, ΔΦ, Δ*v* and Δ*P* are regarded as the input for the combined filter, and the output of the combined filter is regarded as the feedback for implementing the navigation correction.

Positioning output by SINS can be expressed as

(21) $$\begin{array}{c} L_{\text{SINS}}=L+\Delta L,\\ \lambda_{\text{SINS}}=\lambda +\Delta \lambda , \end{array}$$

where *L*_SINS_ is latitude of the shearer output by SINS, *λ*_SINS_ is longitude of the shearer output by SINS, and *L* and *λ* are the real value of latitude and longitude, respectively.

Similarly, positioning output by WSN can be expressed as

(22) $$\begin{array}{c} L_{\text{WSN}}=L-\frac{\Delta R_{y}}{R_{N}},\\ \lambda_{\text{WSN}}=\lambda -\frac{\Delta R_{x}}{R_{E}\cos L}, \end{array}$$

where Δ*Rx* is eastward measuring range error and Δ*R*_*y*_ is northward measuring range error. Take Δ*P* as observation; then location observation equation of the shearer can be determined by

(23) $$\begin{array}{c} Z_{k}\left(t\right)=\left[\begin{array}{c} \left(L_{\text{SINS}}-L_{\text{WSN}}\right)R_{N}\\ \left(\lambda_{\text{SINS}}-\lambda_{\text{WSN}}\right)R_{E}\cos L \end{array}\right]=\left[\begin{array}{c} R_{N}\Delta L+\Delta R_{y}\\ R_{E}\Delta \lambda \cos L+\Delta R_{x} \end{array}\right]. \end{array}$$

Another kind of form is

(24) $$\begin{array}{c} Z_{k}\left(t\right)=J_{k}\left(t\right)X_{k}\left(t\right)+V_{k}\left(t\right),\\ J_{k}\left(t\right)=\left[O_{2\times 5},\mathrm{diag}\left[R_{N},R_{E}\cos L\right],O_{2\times 3}\right],\\ V_{k}\left(t\right)=\left[\Delta R_{y},\Delta R_{x}\right]. \end{array}$$

## 4. Simulation Analysis

We simulate the shearer position strategy based on SINS for position accuracy performance; the initial preset values are set as follows.

The earth radius *R* = 6378.16 km, the earth rotation rate *ω* = 7.2916 × 10^−5^ rad/s, gravitational acceleration *g* = 9.78 m/s^2^, initial bias of the linear accelerometer is ac = 9.78 × 10^−5^ m/s^2^, and the random drift ar = 4.89 × 10^−5^ m/s^2^. Initial bias of the gyroscope is pc = 0.02 deg and the random drift is pr = 0.01 deg; the latitude is *φ* = 45 deg, and the measured velocity of random noise is vr = 0.05 m/s. the shearer initial attitude angle is Φ = [0 0 0]^*T*^, the initial velocity is *V*_0_ = [0 0 0]^*T*^, and the initial displacement is *S*_0_ = [0 0 0]^*T*^.

**Figure 7:**
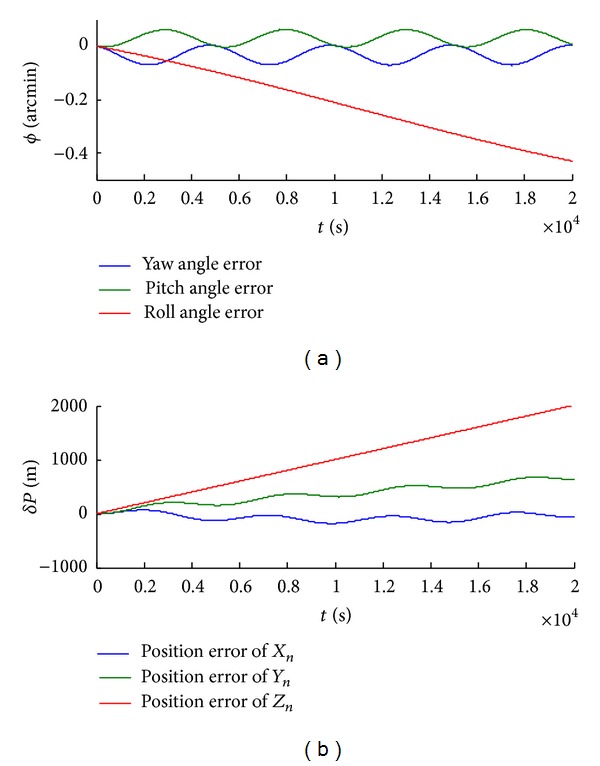
The shearer attitude angle and position biases without real-time calibration.

**Figure 8:**
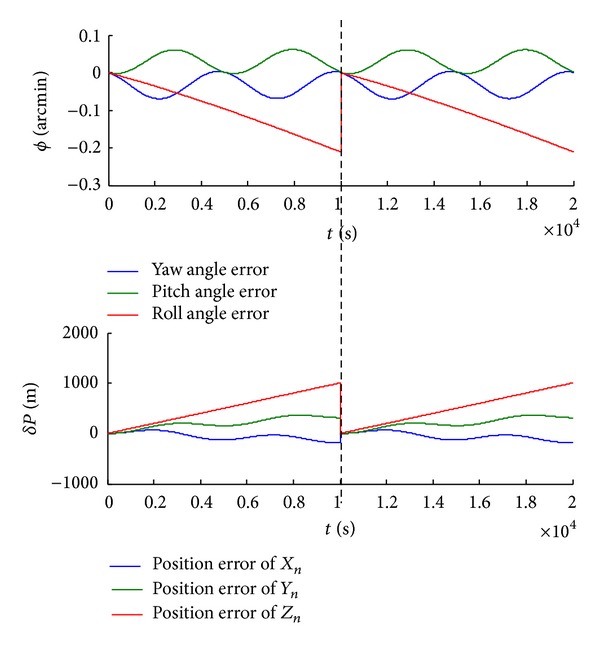
The shearer attitude angle and position biases with one-time calibration.

**Figure 9:**
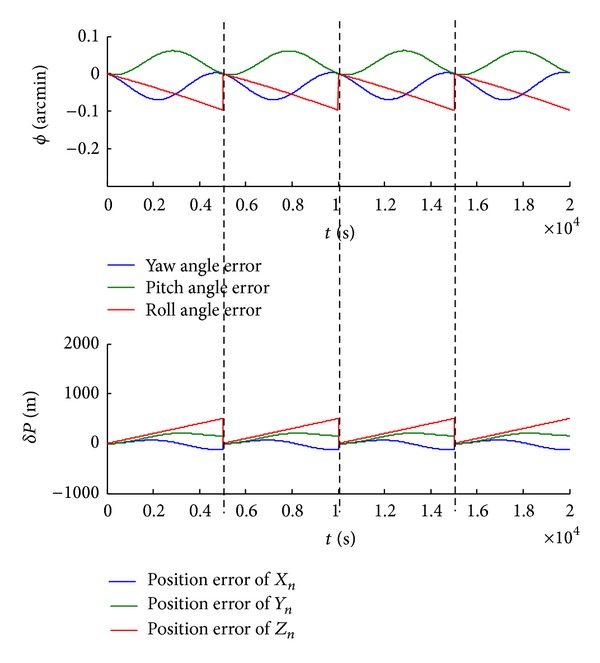
The shearer attitude angle and position biases with three-time calibrations.

(1) We obtain the shearer attitude angle and position bias without real-time calibration. As shown in Figure 7, the shearer roll angle is dispersed because of accumulated error. And the maximum offset is −0.45 arcmin. Meanwhile, the shearer position is dispersed in *X*_*n*_, *Y*_*n*_, and *Z*_*n*_ and the maximum offset is 2000 mm.

(2) In contrast, we real-timely calibrate the SINS of the shearer. Results show that once calibration reduces the error half the original attitude angle, while calibrations of three times reduce one-quarter, as shown in Figures 8 and 9.

Simulation shows that calibration of SINS satisfies navigation accuracy for shearer position. As GPS is unsuitable in the underground, a new technology for calibration is adopted for calibration. Analysis by synthesis, we use infrared plus, in addition, the calibration count is determined depending on the length of the working face.

## 5. Experimental Researches

**Figure 10:**
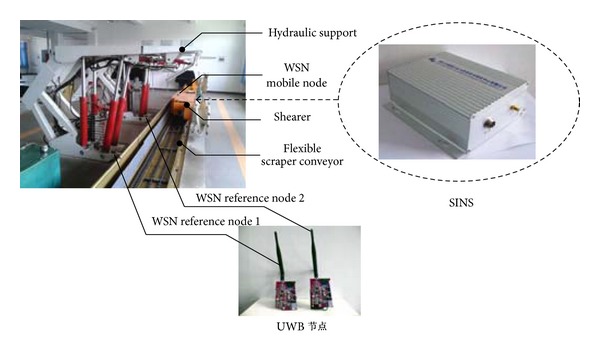
Test platform of SINS/WSN for the shearer.

Experimental platform is built by the ratio of 1 : 3 scale relative to the actual working condition, which mainly includes shearer, hydraulic support, scraper conveyor, and SINS module, as shown in Figure 10.

**Figure 11:**
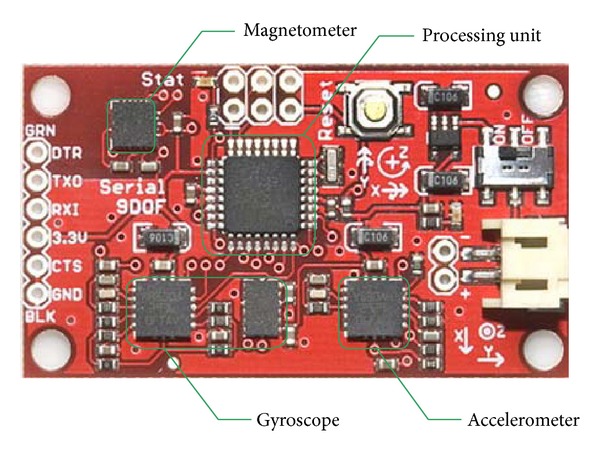
SINS used in the experiment.

**Figure 12:**
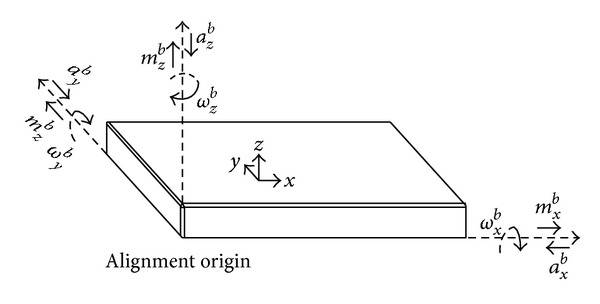
SINS module of the shearer.

As shown in Figures 11 and 12, SF9DOF type SINS is used in the experiment, which includes an acceleration, a magnetometer, and two gyroscopes. This type of the SINS has been used for unmanned aerial vehicle and automatic car driving successfully. Triaxial acceleration sensor is used in the linear accelerometer (ADXL345) and outputs *a*_*x*_^*b*^, *a*_*y*_^*b*^, and *a*_*z*_^*b*^ in three axes. Meanwhile, two-axis gyroscope (LPR530AL) and single-axis gyroscope (LY530AL) are used to obtain the shearer angle velocity. LPR530AL outputs *ω*_*x*_^*b*^ and *ω*_*y*_^*b*^, while *ω*_*z*_^*b*^ is measured by LY530AL. Triaxial magnetometer (HMC5843) are used for shearer attitude compensation and outputs *m*_*x*_^*b*^, *m*_*y*_^*b*^, and *m*_*z*_^*b*^.

Other configurations are as follows: processor is ATMEGA328; data transmission uses serial communication and the baud rate is 57600 bit/s; the sampling period is 0.01 s.

### 5.1. Static Tracking Test of Attitude Angle

In the process of testing, attitude tracking conditions are shown in Table 1.

**Figure 13:**
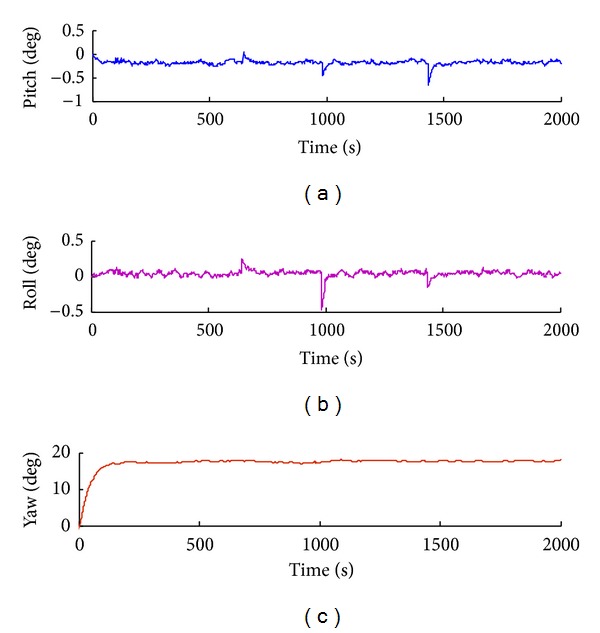
Attitude tracking when pitch angle *β* = 0, roll angle *φ* = 0, and yaw angle *α* = 17°.

**Figure 14:**
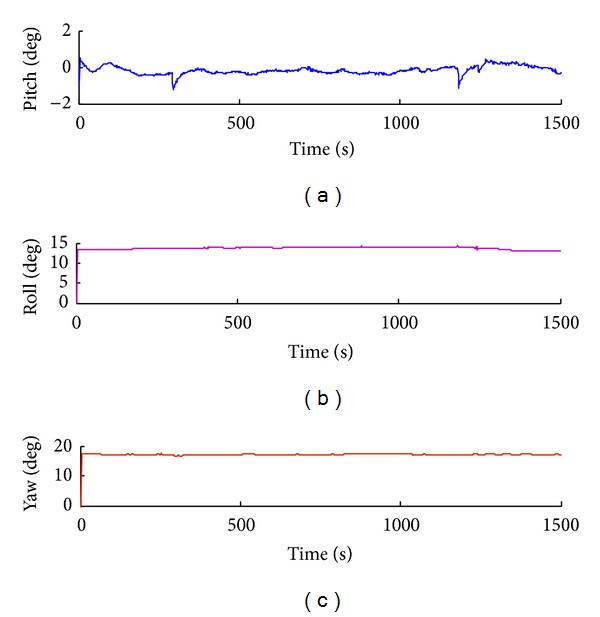
Attitude tracking when pitch angle *β* = 0, roll angle *φ* = 14°, and yaw angle *α* = 17°.

**Figure 15:**
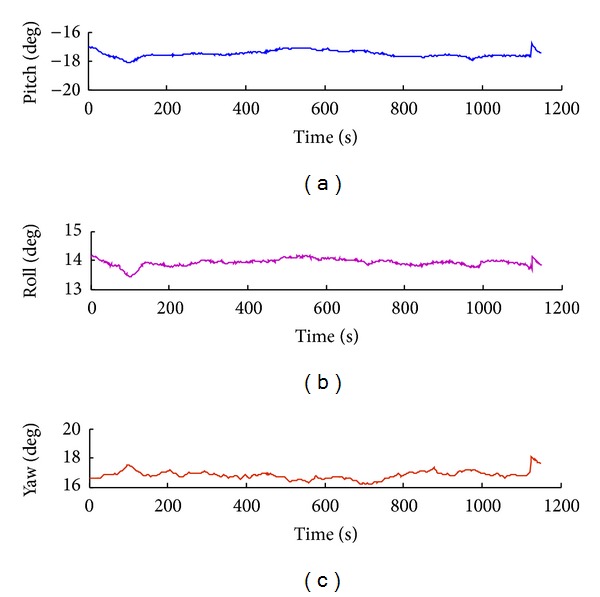
Attitude tracking when pitch angle *β* = −17°, roll angle *φ* = 14°, and yaw angle: *α* = 17°.

**Figure 16:**
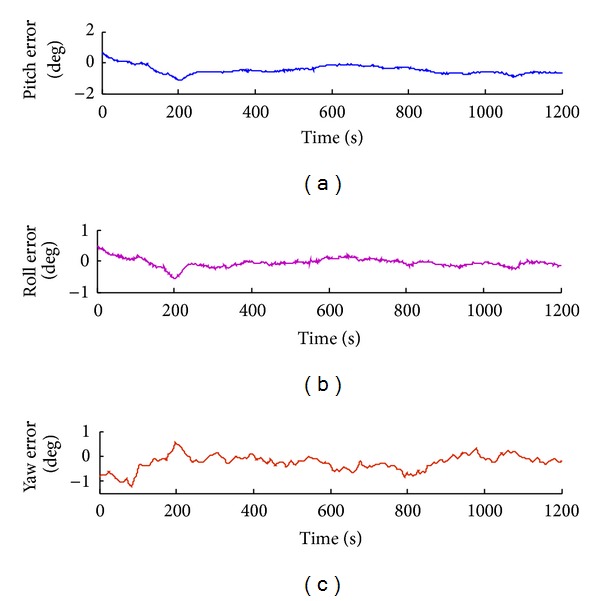
Attitude tracking error when pitch angle *β* = −17°, roll angle *φ* = 14°, and yaw angle: *α* = 17°.

Attitude tracking errors such as pitching angle error, yaw angle error, and roll angle error are obtained by tracking experiment, as shown in Figures 13, 14, 15, and 16.

### 5.2. Dynamic Tracking Test of Attitude Angle

**Figure 17:**
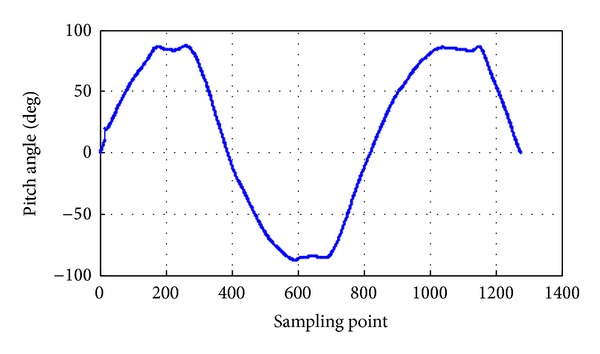
Dynamic tracking of pitch angle.

**Figure 18:**
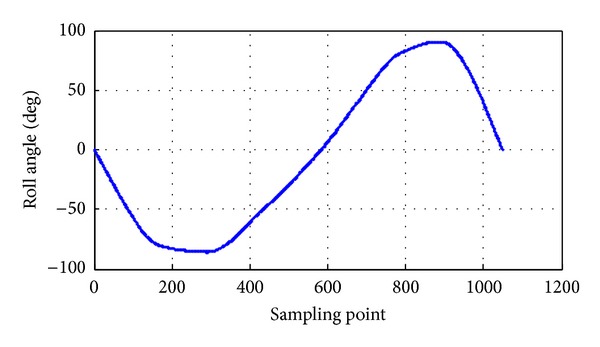
Dynamic tracking of roll angle.

**Figure 19:**
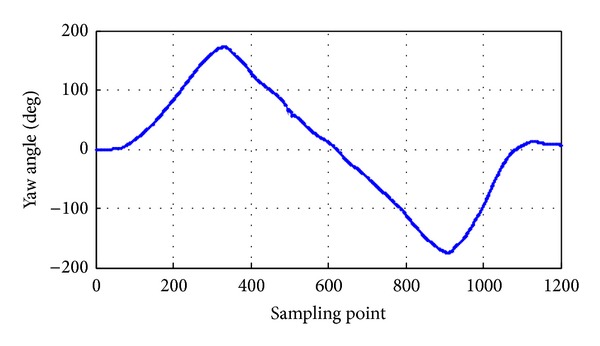
Dynamic tracking of yaw angle.

**Figure 20:**
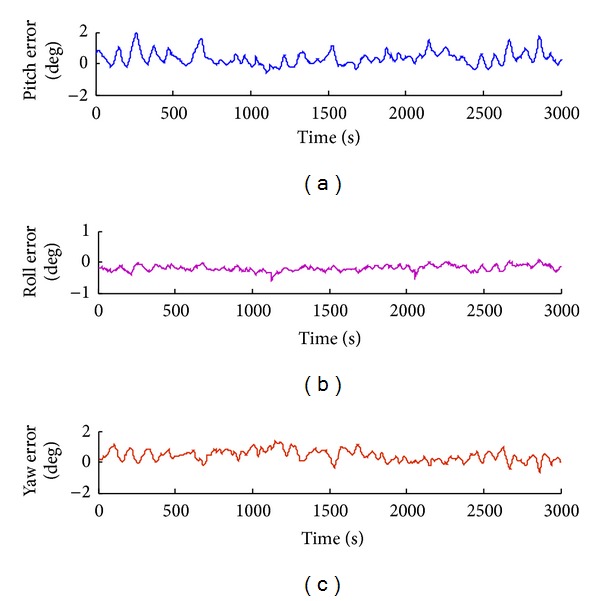
Attitude dynamic tracking error of the shearer with inertial navigation position system.

**Figure 21:**
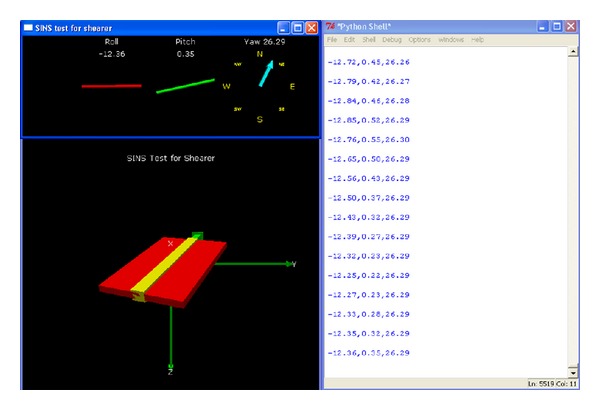
PC interface in the experimental process.

The pitch angle rate is 0.7 rad/s, and the pitch angle range is between −90° and 90°; the pitch angle tracking is shown in Figure 17. The roll angle rate is 0.5 rad/s, and the roll angle range is between −90° and 90°; the roll angle tracking is shown in Figure 18. The yaw angle rate is 0.5 rad/s, and the yaw angle range is between −180° and 180°; the yaw angle tracking is shown in Figure 19. Attitude dynamic tracking error is shown in Figure 20, and PC interface is shown in Figure 21.

### 5.3. Tracking Test of Position

**Figure 22:**
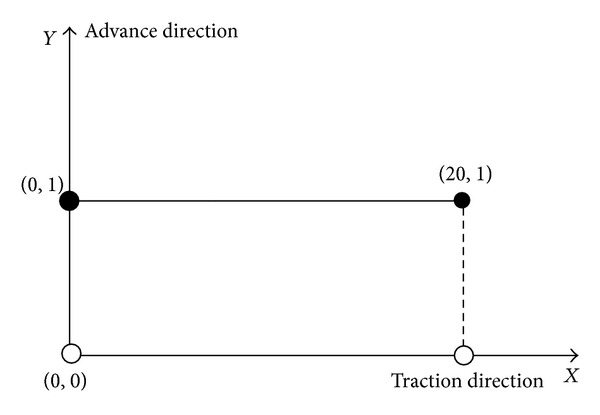
Lines predetermined trajectory of the shearer.

**Figure 23:**
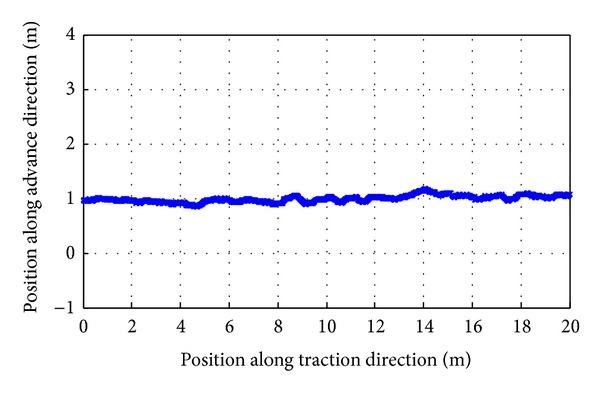
Linear trajectory tracking of the shearer based on SINS/WSN.

**Figure 24:**
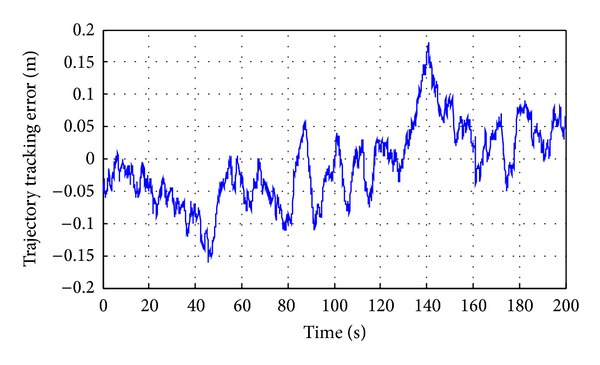
Linear trajectory tracking error of the shearer.

*(1) Linear Motion Trajectory Tracking of the Shearer*. Lines predetermined trajectory has been set, as shown in Figure 22. *Y* axis is set to 1, and the initial position *P*_0_ = (0,1), the termination position *P*_2_ = (20,1). The results are shown in Figures 23 and 24.

**Figure 25:**
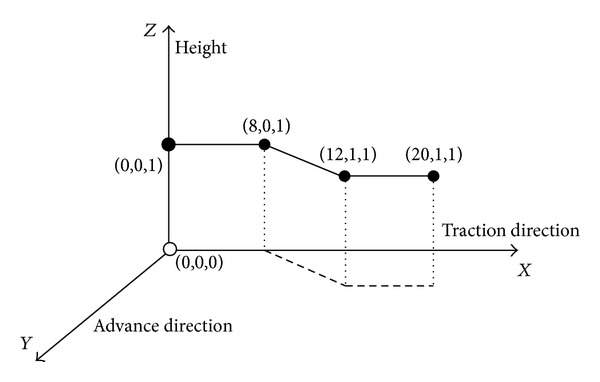
Broken line predetermined trajectory of the shearer.

**Figure 26:**
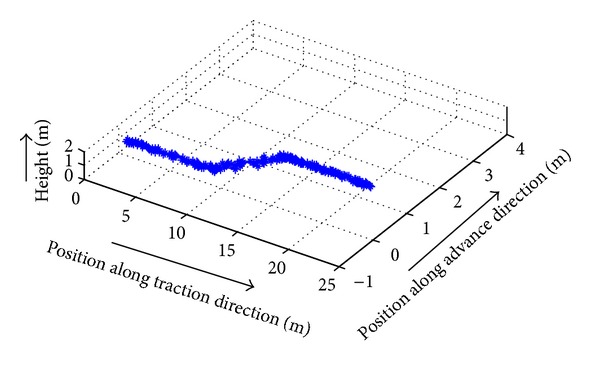
Broken line trajectory tracking of the shearer basing on SINS/WSN.

**Figure 27:**
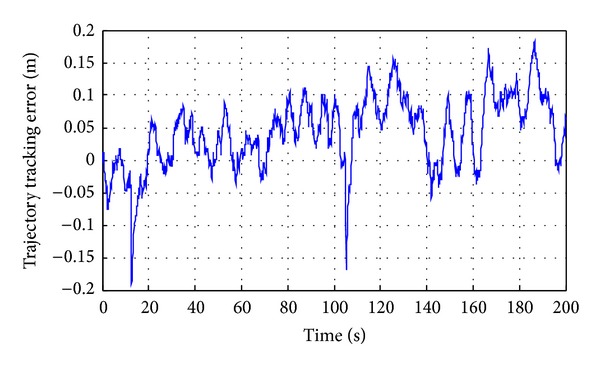
Broken line trajectory tracking error of the shearer.

*(2) Broken Line Motion Trajectory Tracking of the Shearer*. Because of oblique cutting feed motion in the working face of coal cutting operation, broken line predetermined trajectory has been set, as shown in Figure 25. Pushing distance along the advance direction is 1 m, and working face length is 20 m. In the process of the test, *Z* axis is set to 1 m, the initial position *P*_0_ = (0,0, 1), oblique cutting feed starting point is *P*_1_ = (8,0, 1), oblique cutting feed ending point is *P*_2_ = (12,1, 1), the termination positioning is *P*_3_ = (20,1, 1), and test time is 200 s. Due to the fact that signal output frequency of SINS is 100 Hz and signal output frequency of WSN is 5 Hz, filtering cycle is set to 0.2 s in order to match parameters of SINS/WSN; the results are shown in Figures 26 and 27.

### 5.4. Result Analysis

- In the process of attitude angle static tracking test, while the pitch angle is *β* = −17°, pitching angle is *φ* = 14°, and yaw angle is *θ* = 17°, the average tracking angles are *β*_avr_ = −17.47°, *φ*_avr_ = 13.94°, and *θ*_avr_ = 16.74°. Mean square errors are corresponding to 0.08, 0.02, and 0.09. Average error ranges are Δ*β* = (−1.13° ~ 0.66°), Δ*φ* = (−0.58° ~ 0.5°), and Δ*θ* = (−1.24° ~ 0.56°), as shown in Figure 16.
- In the process of attitude angle dynamic tracking test, the dynamic tracking angle error ranges are, respectively, Δ*β* = (−0.63° ~ 1.96°), Δ*φ* = (−0.62° ~ 0.06°), and Δ*θ* = (−0.65° ~ 1.35°), and mean square errors are corresponding to 0.1894, 0.0082, and 0.1275.
- In the process of linear motion trajectory tracking, position error is *P*_error1_ = (−0.16 m ~ 0.18 m), the average residual rate is 6.7%, and the confidence coefficient is 93.3%, as shown in Figure 24.
- In the process of broken line motion trajectory tracking, position error is *P*_error2_ = (−0.191 m ~ 0.183 m), the average residual rate is 8.9%, and confidence coefficient is 91.1%, as shown in Figure 27.

Results show that there is no divergence for positioning error, attitude tracking error is less than 0.7°, position tracking error is less than 0.2 m, which can satisfy the shearer positioning precision.

## 6. Conclusions

- The biggest feature of SINS is based on the vector itself inertial positioning information. Compared to the other traditional ways, this method has better autonomy and reliability.
- As we cannot use GPS for SINS real-time correction in the coalmine, this paper proposed the way of external correction by WSN. Simulation result shows that the accumulated error of SINS is reduced effectively by external fixed point correction, and system precision is improved.
- According to the experimental analysis, integrated positioning system based on SINS/WSN can track attitude and position of the shearer in real time, which is suitable for the shearer positioning system.

## Figures and Tables

**Table 1:** Static test conditions of the shearer attitude angle.

	Condition 1	Condition 2	Condition3
Yaw angle	17°	17°	17°
Roll angle	0	14°	14°
Pitch angle	0	0	−17°
